# Synthesis of Hierarchical Nanoporous Microstructures via the Kirkendall Effect in Chemical Reduction Process

**DOI:** 10.1038/srep16061

**Published:** 2015-11-10

**Authors:** Ling Gao, Chao Pang, Dafang He, Liming Shen, Arunava Gupta, Ningzhong Bao

**Affiliations:** 1 State Key Laboratory of Material-Oriented Chemical Engineering, Nanjing Tech University (Former Name: Nanjing University of Technology), Jiangsu, Nanjing 210009, P.R. China; 2 Center for Materials for Information Technology (MINT), The University of Alabama, Tuscaloosa, AL 35487, USA

## Abstract

A series of novel hierarchical nanoporous microstructures have been synthesized through one-step chemical reduction of micron size Cu_2_O and Co_3_O_4_ particles. By controlling the reduction time, non-porous Cu_2_O microcubes sequentially transform to nanoporous Cu/Cu_2_O/Cu dented cubic composites and hollow eightling-like Cu microparticles. The mechanism involved in the complex structural evolution is explained based on oxygen diffusion and Kirkendall effect. The nanoporous Cu/Cu_2_O/Cu dented cubic composites exhibit superior electrochemical performance as compared to solid Cu_2_O microcubes. The reduction of nonporous Co_3_O_4_ also exhibits a uniform sequential reduction process from nonporous Co_3_O_4_ to porous Co_3_O_4_/CoO composites, porous CoO, porous CoO/Co composites, and porous foam-like Co particles. Nanoscale channels originate from the particle surface and eventually develop inside the entire product, resulting in porous foam-like Co microparticles. The Kirkendall effect is believed to facilitate the formation of porous structures in both processes.

The properties of functional materials are generally dependent on their microstructure. Volume defects (voids) commonly occur during materials synthesis are usually not desirable. However, they can be exploited for practical use in specific functional materials^1^,^2^. Porous structured materials have a wide range of potential applications in areas such as separation, catalysis, energy conversion and storage, targeted drug delivery, etc.^3^,^4^,^5^. The fabrication of solid materials with controllable porous microstructures has been intensively investigated over decades and various manipulation strategies, from templated methods^6^,^7^,^8^,^9^,^10^ to self-templated methods^11^,^12^,^13^,^14^,^15^,^16^,^17^, have been developed. Due to the exclusive template-free feature, self-templated methods such as Ostwald ripening, galvanic replacement, Kirkendall effect, etc., have become fairly common strategies for producing novel controllable porous structures^11^,^12^,^13^,^14^,^15^,^16^,^17^. Although there are numerous reports of processes for morphology control of porous structures, most of them are focused on the formation of novel hollow metals, metal oxides, and metal chalcogenides^11^,^15^,^18^. There are only a few reports on porous metallic structures obtained through reduction of their respective compounds. Recently, porous Si has been prepared from porous silica precursor through magnesiothermic reduction for use in lithium-ion batteries^19^. However, due to the complexity of the mechanism and the difficulty of controlling the compositional and structural transformations, it is unlikely that this reduction process can be extended for the synthesis of other porous structured materials.

Thus far the preparation of porous metallic structures has been mostly limited to noble metals including Au, Ag, Pt, and their alloys, both for fundamental research and technological applications^9^,^15^. The formation of porous structures during the oxidation process is often explained in terms of the Kirkendall effect, which has been utilized for the formation of unique porous nanostructures^20^,^21^,^22^,^23^. But the mechanism is more complicated in the metal oxide reduction process where several factors, such as the driving force of the metal-oxygen bond cleavage, the path of oxygen outward diffusion, metal-oxide interface shift, crystal lattice deformation and reorientation, etc., play key roles at different stages but cannot be *in-situ* monitored and controlled.

Cu_x_O is one of the most widely used catalysts because of its high activity and selectivity for many oxidation/reduction reactions^24^,^25^. The reduced metal oxides usually exhibit higher catalytic activity than the pure stoichiometric CuO. Cu_x_O is also an essential component in copper-oxide-based high-Tc superconductors, wood protection, and antimicrobial products^26^,^27^,^28^,^29^. Therefore, the reduction of Cu_x_O, particularly under H_2_ or CO reduction atmosphere, has been widely investigated. Although the mechanism at the atomic level is still unresolved, the studies thus far indicate that O vacancies play a key role in the reduction of Cu_x_O. These research results have inspired us to utilize the reduction processes to synthesize porous Cu-based materials with novel architecture.

We have developed a simple solution-phase method for fabricating nanoporous Cu-based microstructures by thermal reduction of solid non-porous Cu_2_O microcubes. Very interesting morphologies, including nanoporous Cu/Cu_2_O/Cu dented cubic composites and hollow Cu with eightling (eight-fold twinning)-like structures are obtained during the reduction process. The product mixture can be readily controlled using this facile one-step process with the reduction time being the only controlling factor. The formation mechanism is discussed based on oxygen diffusion and the Kirkendall effect. Attributed to its porous structure, the nanoporous Cu/Cu_2_O/Cu dented cubic composites exhibit superior structural and electrochemical performance over solid Cu_2_O microcubes. To demonstrate the generality of the reduction process for preparing nanoporous structures, we have also carried out the solution-phase reduction of Co_3_O_4_. Sequential Co-based porous products, with tunable structure and chemical composition, are obtained by simply adjusting the reduction time. Our work demonstrates the feasibility of fabricating porous structures via reduction process and will likely inspire interest in the preparation of practical porous materials by this facile method and further investigation of the formation mechanism.

## Results and Discussion

**Figure 1:**
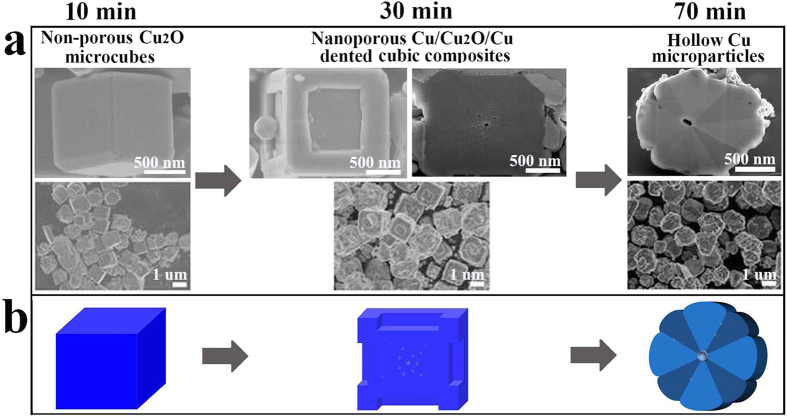
SEM and schematic of the Cu-based microstructures. (**a**) SEM images and (**b**) schematic illustration of the Cu-based microstructures formed during the solution-phase reduction of Cu_2_O at different reaction times (10, 30, and 70 min).

Figure 1a show the SEM images of all the Cu-based products obtained from solution after different reaction time. The starting material of the reduction process is in the form of non-porous solid Cu_2_O microcubes with an average size of about 1.4 μm. FIB-FESEM images (Figure S1) of the cross sections of a typical Cu_2_O microcube confirm its nonporous inner structure. After 30 min of reduction, the non-porous Cu_2_O microcubes transform to dented cubic composites with similar size, as shown in Figure S2 of the large-area SEM image of the dented cubic composites. The cross-section SEM image of a typical dented cubic composite indicates that numerous nanopores (less than 30 nm) are generated inside the cubes with the larger pores being closer to the cube center (Figure S3). With increasing of the aging time to 50 min, the surface Cu particles merge and agglomerate at the surface (Figure S4). After 70 min of reduction, the dented microcubes transform to irregular microparticles. The cross-section SEM image clearly shows eightfold twinning-like segments surrounding a large pore (about 100 nm) in the center of the microparticles, forming hollow structure. Figure 1b shows the schematic illustration of the compositional, structural, and morphological evolution of the Cu-based products obtained at different reduction time. The eightling-like structure is already formed inside the nanoporous dented microcubes, as will be discussed later.

**Figure 2:**
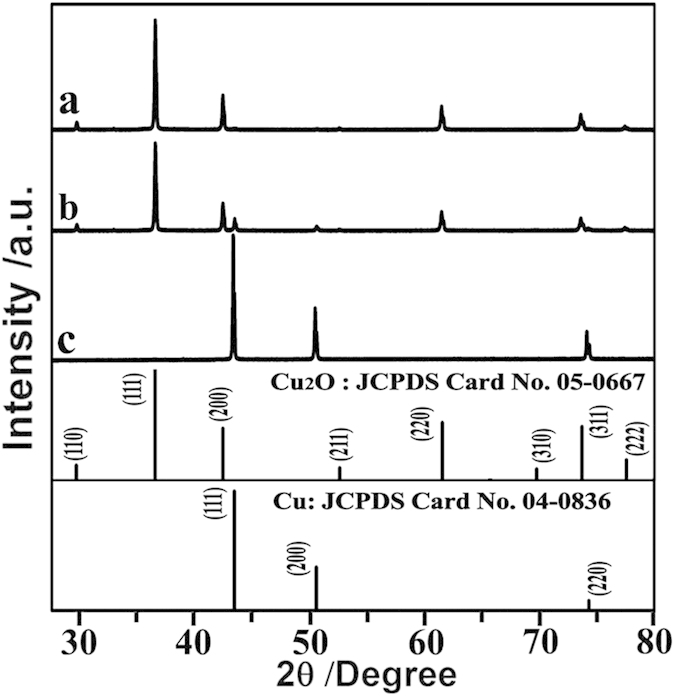
XRD spectra of the Cu-based microstructures formed during the solution reduction of Cu_2_O at different reaction times. (**a**) 0 min for non-porous Cu_2_O microcubes, (**b**) 30 min for nanoporous Cu/Cu_2_O/Cu dented cubic composites, and (**c**) 70 min for hollow eightling-like Cu microparticles.

**Figure 3:**
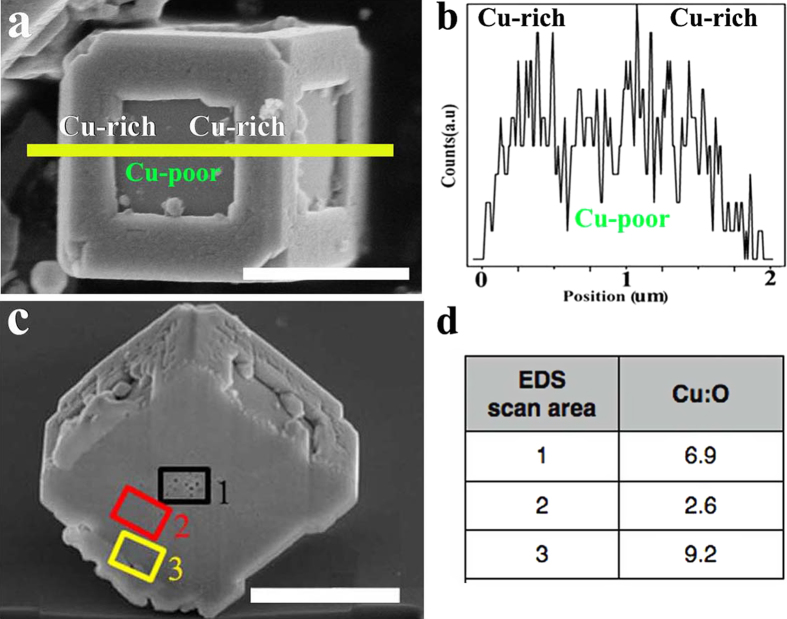
(a–b) The surface and (c–d) inner chemical compositions of the nanoporous Cu/Cu_2_O/Cu dented cubic composites formed after 30 min reduction. (**a**) SEM image and (**b**) line-scan EDS profile of the surface of a typical nanoporous Cu/Cu_2_O/Cu dented cubic composites. (**c**) FIB-FESEM cross section image of the same sample, showing the inner nanoporous structure. (**d**) Cu/O ratio of the three areas marked in (**c**) as determined by area-scan EDS. The scale bars represent 1 um.

The crystal phase of the above three products have been examined using XRD as shown in Fig. 2. The XRD pattern of the non-porous Cu_2_O microcubes (10 min, Fig. 2a) can be indexed to pure cuprous oxide (Cu_2_O). The cubic shape of the Cu_2_O crystals suggests that growth of the (111) face is relatively fast^30^,^31^,^32^. The diffraction peaks of the nanoporous Cu/Cu_2_O/Cu dented cubic composites (30 min, Fig. 2b) can be indexed to a mixture of face-centered (fcc) cubic Cu and Cu_2_O, indicating partial reduction of Cu_2_O to Cu. The diffraction peaks from the hollow eightling-like microparticles belong to pure *fcc* Cu, confirming that Cu_2_O is completely reduced to Cu after 70 min. Due to the coexistence of Cu and Cu_2_O in the nanoporous dented cubic composites, we carried out energy dispersive X-ray spectroscopy (EDS) analysis on this sample to obtain the chemical composition profile. For this purpose, a surface line-scan EDS is first carried out on a typical sample, as shown in Fig. 3a. The generated elemental line profile (Fig. 3b) indicates that the dented cubic composites are relatively Cu-rich at the edges and Cu-poor at the dented regions. To understand the internal composition profile, we analyzed three areas from the cross section of the same sample by area-scan EDS, as shown in Fig. 3c. The area 3, i.e. the edge area, has a Cu/O ratio of 9.2. It is apparently Cu-rich, in agreement with the result from the surface composition analysis. The area 1, i.e. the nanoporous center has a Cu/O ratio of 6.9, also indicating relatively Cu-rich. On the other hand, the area 2 has a much lower Cu/O ratio of 2.6, close to that in Cu_2_O. Therefore, the chemical compositional profile of the porous dented microcubes is suggested to be a nanoporous Cu/Cu_2_O/Cu sandwich-structured composite.

**Figure 4:**
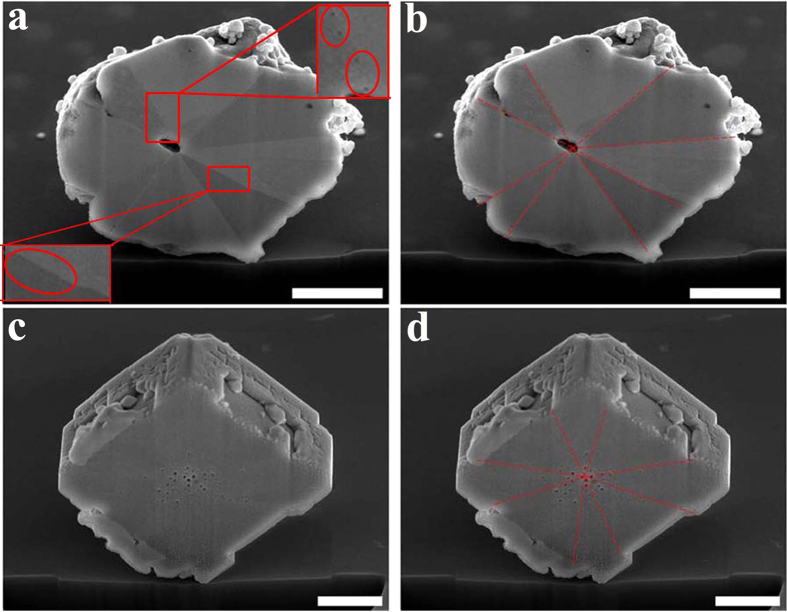
Cross section SEM images of a typical particle. (**a–b**) Cross section SEM images of a typical hollow eightling-like Cu microparticle formed after 70 min. The dash lines in b indicate the clear twinning interfaces. (**c–d**) The SEM images of the cross section of a typical nanoporous Cu/Cu_2_O/Cu dented cubic composite particle formed after 30 min. The dashed lines in d show the possible twinning interfaces for oxygen diffusion path. The scale bars represent 500 nm.

Besides the sandwich structure, the nanoporous Cu/Cu_2_O/Cu dented cubic composites also exhibit another remarkable feature, i.e. an eightfold twinning-like structure. Crystal twinning is the result of an accidental departure from equilibrium during crystal growth that results in crystals separated by twin boundaries^33^. For example, during explosion, the extreme stress can result in the formation of twinning structures in a *fcc* metal^34^. Nanotwinned Cu exhibits ultrahigh strength attributed to coherent internal boundaries which obstructs dislocation motion^34^,^35^,^36^,^37^. If multiple repeated twins are not parallel they are called cyclic twins. Cyclic twinning structures are commonly observed in naturally occurring minerals^38^. Eight-fold cyclic twinning structure is also referred to as eightling structure. As shown in Fig. 4a,b, the cross section of a typical hollow Cu microparticle clearly displays an eightling-like structure, with eight-fold repeating sectors surrounding the central hole. It is not clear if this repeating pattern can fit the strict definition of crystal twinning or not. The twin boundaries are highlighted as red dashed lines in Fig. 4b. It is worth pointing out that a few tiny pores (circled out in Fig. 4a) are observable along some twin boundaries. Actually, the eightling-like repeating pattern begins to be faintly observable in the nanoporous Cu/Cu_2_O/Cu dented cubic composites, as shown in Fig. 4c,d. Close inspection reveals that a number of the nanopores coincide with the twinning boundaries. We thus speculate that the interfacial boundaries between the repeating sectors act as out diffusion pathways for O leading to the formation of the nanoporous sandwich dented cubic composites and subsequent hollow eightling-like microparticles. The formation of porous structure in the reduction reaction can be explained by the Kirkendall effect.

**Figure 5:**
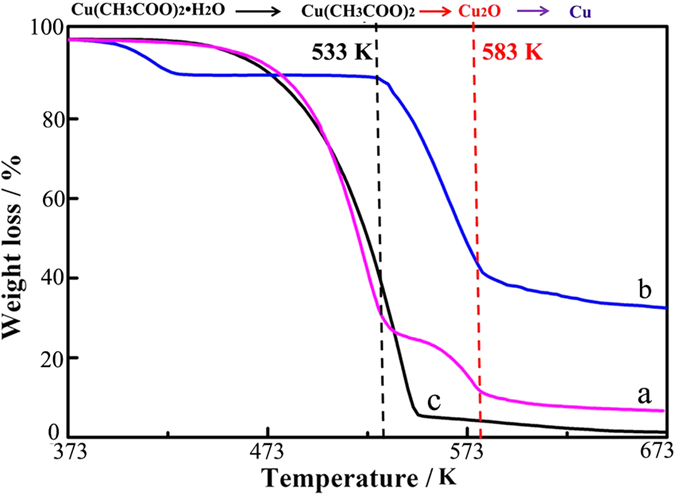
TGA curves of the mixture in N_2_. TGA curves in N_2_ for (**a**) mixture of Cu(CH_3_COO)_2_·H_2_O and oleic acid, (**b**) pure Cu(CH_3_COO)_2_·H_2_O, and (**c**) pure oleic acid.

In our experiments, Cu(CH_3_COO)_2_·H_2_O is dissolved in oleic acid at a relative low temperature of 343 K to avoid formation of the Cu-oleate (Cu^2+^(C_18_H_33_O_2_)–_2_) complex. As shown in the TGA profile (Fig. 5), there is no reaction between copper ions and oleic acid below 533 K except for the evaporative loss of oleic acid and loss of the water of crystallization of Cu(CH_3_COO)_2_·H_2_O. 1-octadecene is a long-chain hydrocarbon with an end alkene group. It is commonly used both as a reducing agent and capping agent in the syntheses of nanoparticles^39^,^40^. On injection of Cu(CH_3_COO)_2_·H_2_O solution into 1-octadecene at 533 K, Cu^2+^ is readily reduced to form insoluble Cu_2_O particles, while the 1-octadecene can be oxidized to ketones, aldehydes and/or hydrocarbons of various chain lengths along with formation of other byproducts such as CO_2_ and H_2_O. Oleic acid, which is used as a solvent, likely also contributes to the thermal decomposition of Cu(CH_3_COO)_2_·H_2_O and is decomposed to form various byproducts. If we just use oleic acid as the solvent we can only obtain Cu particles instead of the formation of the intermediate Cu_2_O products (Figure S5). The excess 1-octadecene and some byproducts likely results in the reduction of Cu_2_O soon after its formation, although the detailed reaction pathway is presently unknown.

The reduction process is generally described by two different kinetic models, namely the “nucleation model” and “interface-controlled model”^41^,^42^. In the “nucleation model”, the reduction process has an induction period (i.e. nucleation period) and possible autocatalysis. The induction period is the rate-determining step. In the “interface-controlled (IC) model”, the reduction process occurs rapidly and a uniform continuous reduced-phase/oxide interface rapidly covers the solid reactant. The reduction rate in this case is proportional to the area of the interface. In our experiment the reduction of Cu_2_O occurs readily at the surface. The XRD results clearly show the continuous chemical conversion from Cu_2_O to Cu, indicating an interface-controlled reduction process. The formation of the nanoporous Cu/Cu_2_O/Cu dented cubic composites is caused by the different oxygen diffusion rates in the external and internal regions of the sample. The external region loses oxygen at a faster rate than it is replenished by internal-oxygen outward diffusion, which results in the external layer being Cu-rich. Moreover, the outward diffusion provides an oxygen concentration gradient inside the sample, which continually drives the internal oxygen outwards according to Fick’s law and thus the central area of the sample also becomes Cu-rich. The outward oxygen diffusion in the intermediate area is compensated by the oxygen from the central area, thus the composition remains close to Cu_2_O. The cuprous Cu_2_O has a lattice constant of 4.2696 Å and *fcc* Cu has lattice parameter of 3.610 Å. Inside the nanoporous Cu/Cu_2_O/Cu dented cubic composites, significant lattice mismatch-induced structural stress will develop between the external Cu and intermediate Cu_2_O with increasing thickness of the external Cu layer. Based on this scenario, we propose that delamination of the region with the highest stress, namely the Cu at the center region of the external surface, occurs, resulting in the dented surface of the cubic composite particles.

**Figure 6:**
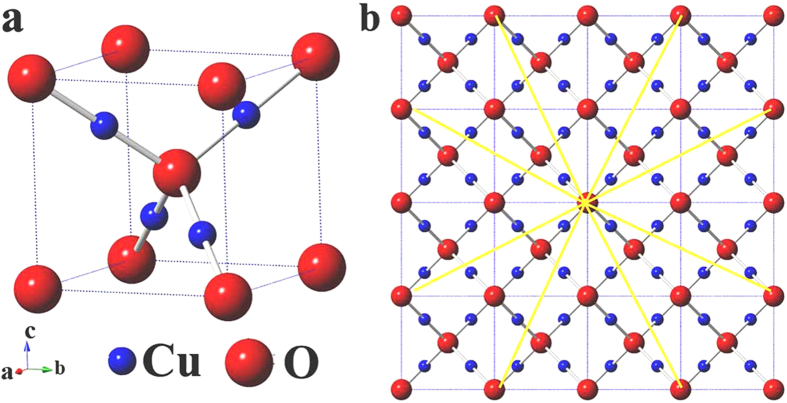
Crystal structure of Cu_2_O. (**a**) Crystal structure of Cu_2_O. (**b**) 2D projection view of 64 Cu_2_O unit cells. Yellow arrows schematically indicate the oxygen outward diffusion pathway. Blue balls represent Cu atoms and red balls represent oxygen atoms.

As per Fick’s law, the direction of movement of atoms is always opposite to the concentration gradient. Therefore, under our thermal reduction conditions, oxygen atoms will radially diffuse out during the reduction of Cu_2_O. As shown in Fig. 6a, the unit cell of Cu_2_O contains 4 Cu atoms and 2 O atoms, with the Cu atoms arranged in a *fcc* sublattice and oxygen atoms occupying the tetrahedral sites. Figure 6b shows the 2D projection view of a crystal lattice with 64 Cu_2_O unit cells. The yellow highlighted arrows schematically indicate the possible oxygen diffusion pathways. Inside a crystal lattice, atomic diffusion occurs either through interstitial sites or defects. During the relatively rapid reduction process of Cu_2_O to Cu, the lattice reconstruction results in abrupt oxygen deficiency and substantial defects are formed inside the crystal structure. Thus, for the shortest mean free path, jump diffusion instead of interstitial diffusion will be kinetically favored. The lattice defects may self-heal or further evolve under the specific thermodynamic conditions of the reaction system. In our case, a unique eightling-like pattern is formed, and the twin boundaries function as high speed channels for oxygen atom migration. The existence of nanopore only appear at the twin boundaries in the eightling-like microparticles substantiates our hypothesis of the high speed oxygen diffusion pathway.

The appearance of the nanopores can be understood based on the Kirkendall effect, as has been done previously to explain metal boundary shift and void formation due to different diffusion rates of elemental components^43^,^44^,^45^. In the present study, the Kirkendall effect occurs at the Cu/Cu_2_O interface, where the outer diffusion of O atoms is much faster than inner diffusion of Cu during this reduction process. The faster outward diffusion of the O atoms as compared to the inward diffusion of the Cu atoms can result in an inward flux of vacancies accompanying the outward O atoms flux to balance the diffusivity difference. When the vacancies become supersaturated, they coalesce into a void to restore equilibrium. While most of the small nanopores gradually coalesce to form larger pores in the eightling-like Cu microparticles, a small number of them still remain along the oxygen diffusion channels. To the best of our knowledge this is the first report of a Kirkendall effect-driven chemical reductive transformation of oxides to metals with tailored nanoporous structures.

**Figure 7:**
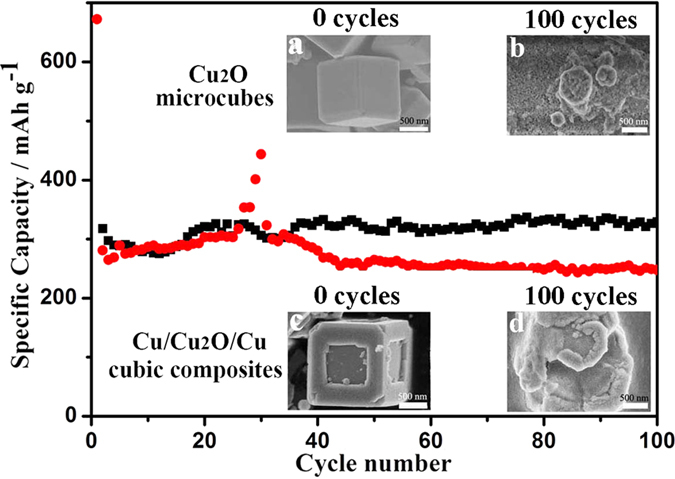
Cycling performances of Li-ion battery and morphology evolution of electrode materials. Cycling performance at a current density of 0.2 C and the corresponding morphology changes of the non-porous Cu_2_O microcubes (red) and nanoporous Cu/Cu_2_O/Cu dented cubic composite (black). The insets are SEM images of (**a,b**) non-porous Cu_2_O microcubes and (**c,d**) nanoporous Cu/Cu_2_O/Cu dented cubic composite (**a,c**) before and (**b,d**) after cycling test. The storage capacities were determined by cycling at 0.2 C rate between 0.02 and 3 V.

Porous hollow structure offer possibilities in material design for applications in catalysis, nanoreactors, energy storage, and drug delivery. To demonstrate the superior property of porous hollow structures over solid structures in energy storage, we compared the performance of the nanoporous Cu/Cu_2_O/Cu dented cubic composites with solid non-porous Cu_2_O microcubes as anode materials. We monitored the structural and morphological changes of the two structures using FE-SEM. As seen in the inset SEM images of Fig. 7, the solid non-porous Cu_2_O microcubes (inset a) are completely broken down to smaller particles (inset b) after 100 lithiation/delithiation cycles. The morphological changes of the nanoporous Cu/Cu_2_O/Cu dented cubic composites (inset c) are less severe, although significant deformation is visible (inset d). This clearly demonstrates that structures with nanopores and surface vacancies can more effectively accommodate the large volume changes of electrodes during the lithiation/delithiation process, as has also been previously reported^3^,^7^. The stable Cu framework also probably contributes to the improved morphological preservation (Figure S6).

**Figure 8:**
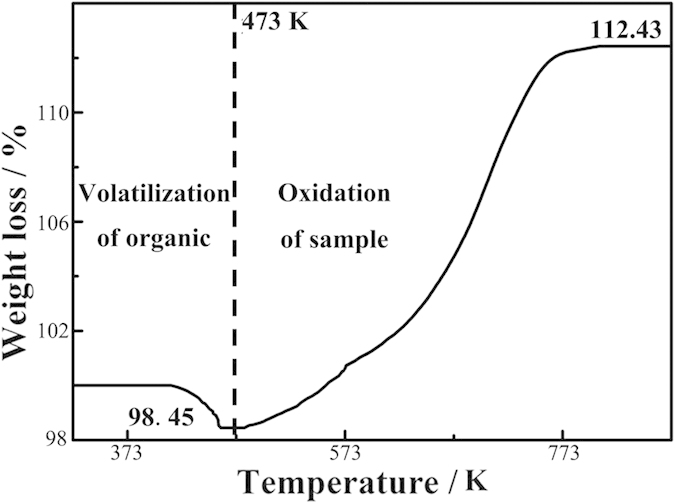
TGA curve for the Cu/Cu_2_O/Cu. TGA curve in air for the nanoporous Cu/Cu_2_O/Cu dented cubic composites heated from room temperature to 873 K at 5 K min^−1^.

Figure 7 also shows the electrochemical performance of both samples cycled at a rate of 0.2 C. The capacity of the sample is calculated from the active material Cu_2_O’s weight which is calculated from the TGA measurement shown in Fig. 8. The initial weigh loss of about 1.55 wt% is caused by evaporation of organics absorbed on the surface of the samples. The chemical reactions occurring during the latter heating process of the sample shown in Fig. 8 are listed as follows: (1) 2Cu+O_2_ = 2CuO; (2) 2Cu_2_O+O_2_ = 4CuO. Assuming the initial sample weight is A and the weight fraction of the Cu_2_O in the sample is X. So the weight fraction of the Cu is 1-X. The equation can be described as follows: 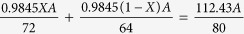 We thus calculate X = 0.7776, the weight fraction of the Cu_2_O in the sample. Thus, the capacities of the samples are calculated from the active material Cu_2_O’s weight (77.76 wt%). As seen in Fig. 7, both samples display stable capacities over 100 cycles, with no sign of any systematic degradation. While the capacity values of the two samples do not show any significant differences, the capacity profile of the solid Cu_2_O microcubes (red line in Fig. 7) exhibits an abrupt peak after about 30 cycles. This can be due to disintegration of the particles that cause a temporary increase in specific surface area. The cycling performance of nanoporous Cu/Cu_2_O/Cu dented cubic composites is superior to that of non-porous Cu_2_O microcubes (Figure S7a). Starting from the fiftieth cycle, the reversible capacity of nanoporous Cu/Cu_2_O/Cu dented cubic composites is stabilized to 320 mAh·g^−1^ after 250 cycles (Figure S7b). In contrast, non-porous Cu_2_O microcubes exhibit an inferior cycling performance, the capacity of non-porous Cu_2_O microcubes gradually decreases from 310 to 250 mAh·g^−1^ after 250 cycles.

**Figure 9:**
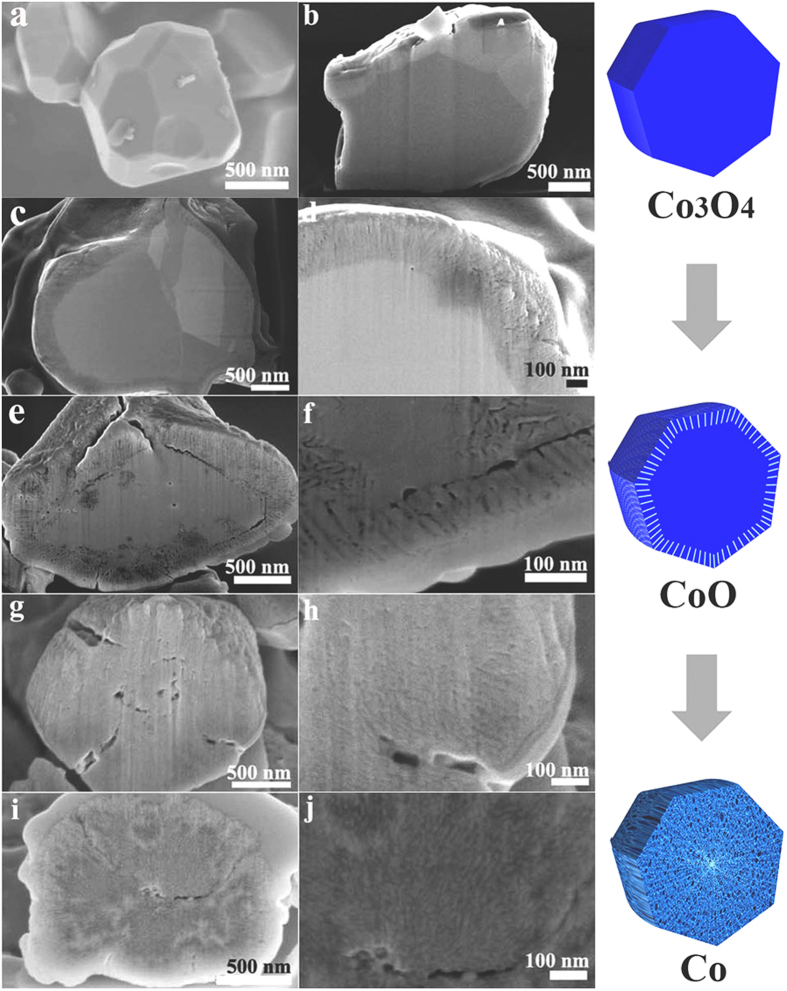
FIB-FESEM images Co-based particles. (**a–b**) Co_3_O_4_, (**c–d**) Co_3_O_4_/CoO nanocomposites, (**e–f**) CoO, (**g–h**) CoO/Co, and (**i,j**) Co prepared by reducing Co_3_O_4_ in oleylamine at 603 K for 0, 20, 50, 80, and 120 min, respectively.

To investigate the generality of the oxygen diffusion pathway and Kirkendall effect-driven nanopore formation, we have also carried out solution reduction of solid Co_3_O_4_ particles dispersed in oleylamine. Similar to oleic acid, oleylamine is a commonly utilized reducing agent and capping agent. Co_3_O_4_ has a normal spinel structure with 32 oxygen ions in one cubic unit cell, while elemental Co has a hexagonal close-packed structure. As compared to Cu_2_O, the reduction of Co_3_O_4_ to Co will be accompanied by significant structural deformation. So the oxygen out-diffusion pathway and Kirkendall effect-driven porous structure during the reduction process should be more readily noticeable. We used non-porous solid Co_3_O_4_ particles as the starting material and a serial of cobalt-based materials, from Co_3_O_4_/CoO, CoO, CoO/Co, to Co, are obtained during the reduction process. The evolution of the porous microstructure and chemical composition transformation during the reaction has been monitored by SEM and XRD, as shown in Fig. 9 and Figure S8.

Figure 9a,b shows that the original Co_3_O_4_ is in the form of non-porous solid particles. After 20 min reduction time, a crust composed of radially aligned nanochannels is formed while the core area of the sample remains solid without any visible pores (Fig. 9c,d). The crust is about 300 nm in thickness. We believe that the crust indicates the initial range of oxygen diffusion, similar to the Cu shell in the Cu/Cu_2_O/Cu composite. As expected, the Co_3_O_4_ crystal lattice undergoes more severe stress during reduction, and abrupt influx of vacancies quickly populate the oxygen diffusion pathway. Figure S8a-b shows the crystal structure change from pure Co_3_O_4_ to Co_3_O_4_/CoO as monitored by X-ray diffraction. The crust is believed to be the CoO layer.

The crust is thicker in the sample reduced for 50 min than the 20 min sample, which indicates continued oxygen out-diffusion. The XRD pattern (Figure S8c) indicates that the original particles are almost completely reduced to CoO. The structural shrinkage going from Co_3_O_4_ to CoO results in small cracks appearing both within the crust and also in the region between the crust and the core, as seen in Fig. 9e,f. As the reaction progresses further (80 min), nanochannel-like oxygen diffusion paths also develop throughout the core (Fig. 9h). Due to the reasonably slower diffusion rate and possible self-healing, the nanochannels in the crust and core-section become similar to each other and the crust-core boundary is eliminated (Fig. 9g). Correspondingly, the sample composition changes from CoO to a mixture of CoO/Co (Figure S8d). For the longest reaction time of 120 min, the sample is transformed into porous foam-like Co particles (Fig. 9i,j and S8e). The cross-section of a typical particle shows numerous nanochannels radially aligned outwards from the center of the particle. The diffraction peaks of foam-like Co particles are relatively broad since the crystallinity is disrupted during the structural evolution. It is interesting that there are only two intermediate phases (Co_3_O_4_ and CoO, CoO and Co) present at different times during the reduction process. This suggests that Co_3_O_4_ undergoes a uniform sequential reduction process to evade any drastic structural transformation. Thus the chemical composition and porous structure of the Co-based products can also be controlled by simply adjusting the reduction duration. Figures S9a-d shows the FT-IR spectra of the Co-based samples. For the original Co_3_O_4_ sample (Figure S9d), no distinct peaks could be observed at 1385 and 1677 cm^−1^, indicating the increase of methyl and carbon double bond functional groups during the reduction of the Co_3_O_4_ in organic solvents for the organic residues.

**Figure 10:**
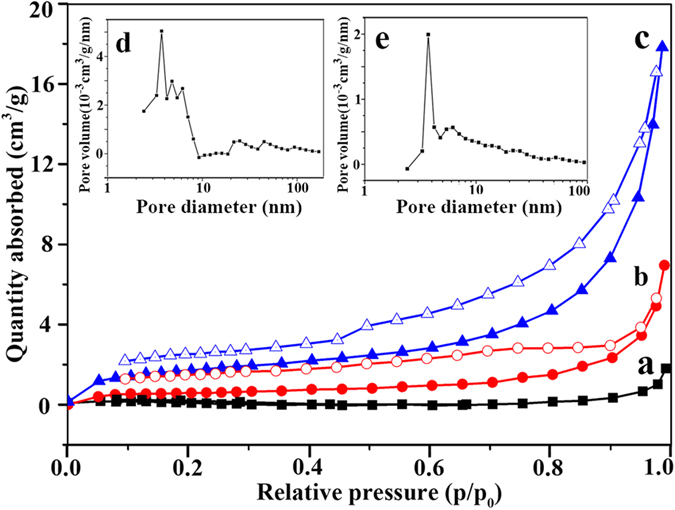
The nitrogen absorption/desorption isotherms for the three different samples. (**a**) the solid Co_3_O_4_, (**b**) CoO particles synthesized for 50 min, and (**c**) Co particles synthesized for 120 min. Insets show the pore size distribution plots of (**d**) the CoO particles and (**e**) Co particles.

The microstructure of the Co-based samples was studied by the nitrogen absorption/desorption isotherms. With increasing of the aging time, more and more O atoms diffuse out from the particle leaving vacancies behind. These vacancies merge and agglomerate to form voids. The hysteresis loops shown in Fig. 10b,c suggest the existence of nanoporous structures, which gives increased surface areas from the non-porous Co_3_O_4_ particles (Fig. 10a) to 1.1 m^2^/g for porous Co_3_O_4_/CoO particles and 5.1 m^2^/g for porous foam-like Co particles. Since the samples are micron-sized particles, the measured surface areas are smaller as compared to nanomaterials. The pore size distribution curves (insets in Fig. 10) calculated by using the BJH method show that the existing mesopores have diameters of 1–4 nm. In contrast, the nitrogen absorption/desorption isotherms of all the Cu-based samples do not show any evidence of porous structure, which is likely due to the melting of surface Cu. The diffusion coefficient of Cu (~10^−10^–10^−8^ nm^2^/s) is far higher than that (~10^−19^–10^−16^ nm^2^/s) of Co. Meanwhile, the melting points of bulk Cu is about 400 °C lower than that of bulk Co^20^, and the melting point of nano-sized Cu has been reported to be much lower (~320 °C for 23 nm Cu and ~224 °C for 13 nm Cu)^46^. Therefore, the initially reduced surface Cu nano-sized layer having much lower melting point results in the rapid movement and melting of initially formed surface Cu nano-sized thin layer, as shown in Fig. 3 and S4. The inner porous structure can only be observed directly on the inner cross section of a cut particle using SEM, as shown in Fig. 3c.

The differences in diffusion coefficients and melting points of Cu and Co metals in reduction reactions also result in the difference in both the reduction conditions and the microstructures of the reduced products. High-temperature solvents including 1-octadecene, oleic acid, and oleylamine are commonly used in the nanoparticle syntheses. Oleylamine has a higher boiling points (~613 K) as compared with that (~573 K) of 1-octadecene. We found that the Co_3_O_4_ particles can only be reduced into Co at higher temperature in oleylamine, which could be due to the higher melting point of Co and stronger reducibility of oleylamine as compared to Cu and 1-octadecene, respectively. Meanwhile, the higher melting point and smaller diffusion coefficient of Co help to maintain the voids and diffusion channels of O atoms, resulting in the formation of nanochannels and porous foam-like structures shown in Fig. 9.

Moreover, the particle size has been found to strongly affect the microstructure of the final products. For example, Yin’s group successfully prepared hollow (~15 nm) nanomaterials by Kirkendall effect^11^, and suggested that the supersaturated vacancy cloud in small nanocrystals is likely to coalesce into a single void. In contrast, the inter-diffusion of large size 30 μm powders with layered composition showed a large volume fraction of pores, but the geometry and distribution of the pores were not uniform because of aggregation and the bulk-like dimension of the particles^47^. Nakamura *et al*. reported failure in the synthesis of hollow PbO since the diffusivity difference (D_Pb_ < D_O_) in PbO does not lead to the formation of vacancy clusters^23^. They also found that the particle size strongly influence the structure of the final product: the final product is hollow if the diameter of Al is <8 nm, in contrast, a core-shell structure can be prepared if the diameter is >8 nm. In these non-reduction reactions, the solid core nanocrystal surface is usually reacted with the reagents to produce a layer of the final product. The direct conversion of the core material to the shell material is therefore hindered by the layer and further reaction will continue by the diffusion of atoms or ions through the interface. If the diffusion rate of the core material is faster than that of the shell material, the preferred outward diffusion of atoms or ions from core material to shell material leads to a net material flux across the nanocrystal interface and simultaneously results in a flow of fast-moving vacancies to the vicinity of the solid-liquid interface. Therefore, hollow voids are formed through coalescence of the vacancies based on nanoscale Kirkendall effect. If the inner diffusion of the shell materials is faster than the outer diffusion of the core materials, the Kirkendall effect led to the formation of voids, such as cracks and pores in the shells^48^.

In summary, we have synthesized nanoporous Cu/Cu_2_O/Cu dented cubic composites and hollow eightling-like Cu microparticles by chemical reduction of solid Cu_2_O microcubes in solution. The chemical composition and morphology of the microstructures have been thoroughly investigated by XRD, EDS, and SEM. The formation mechanism is discussed based on possible oxygen out diffusion and the Kirkendall effect. To the best of our knowledge, this is the first report of the Kirkendall effect in a reduction process leading to the synthesis of porous microstructures. As compared to solid non-porous Cu_2_O microcubes, nanoporous Cu/Cu_2_O/Cu dented cubic composites exhibit better electrochemical performance. The reduction of Co_3_O_4_ also exhibits a uniform sequential reduction process from Co_3_O_4_ to (Co_3_O_4_ and CoO), CoO, (CoO and Co), and Co. Nanoscale channels, which originate from the particle surface and eventually develop in the bulk, result in foam-like Co microparticles. The reduction of Co_3_O_4_ confirms the generality of the reduction process for tunable (both composition and structure) synthesis of nanoporous microstructures. This simple approach augments other methods for fabricating porous metal oxide and metallic structures.

## Methods

### Chemicals

Oleic acid (C_18_H_34_O_2_, analytical reagent), oleylamine (C_18_H_37_N, analytical reagent), and 1-octadecence (C_18_H_36_, >90%) were purchased from Aldrich Co., Ltd. Copper acetate (Cu(CH_3_COO)_2_·H_2_O, analytical reagent), cobalt oxide (Co_3_O_4_, analytical reagent), hexane (C_6_H_14_, analytical reagent), and ethanol (C_2_H_6_O, analytical reagent) were obtained from Shanghai Chemical Co., Ltd. All chemicals were used as-received without any further purification.

### Synthesis of non-porous Cu_2_O microcubes

Cu_2_O microcubes, the starting material for the reduction process, were obtained via the solution-phase thermal decomposition of copper acetate (Cu(CH_3_COO)_2_·H_2_O). First, 40 mmol Cu(CH_3_COO)_2_·H_2_O was dissolved in 30 mL oleic acid (C_18_H_34_O_2_) with continuous stirring at 343 K for 4 h until the solution became clear. Then, 5 mL of the solution was rapidly injected into 25 mL of 1-octadecene in a three-neck flask at 533 K under N_2_ atmosphere with continuous stirring. The reaction solution was maintained at 533 K for 10 min to enable complete decomposition of (Cu(CH_3_COO)_2_·H_2_O) to Cu_2_O before being cooled down to room temperature. Finally, the product Cu_2_O was precipitated using a mixture of hexane and ethanol and then collected via centrifugation.

### Reduction of non-porous Cu_2_O microcubes

After the formation of non-porous Cu_2_O microcubes, the reaction solution was kept at 533 K for an additional 30 min or 70 min for the reduction of Cu_2_O microcubes to form nanoporous Cu/Cu_2_O/Cu sandwich cubic composites or porous hollow eightling-like Cu microparticles, respectively. Thus, the synthesis of Cu_2_O microcubes and the reduction products can be achieved using the same starting reaction solution merely by controlling the reaction time. The reduction products were also precipitated with a mixture of hexane and ethanol and then collected via centrifugation.

### Reduction of Co_3_O_4_ in Oleylamine

In a typical chemical reduction of Co_3_O_4_, 0.5 g Co_3_O_4_ was dispersed in 5 mL oleylamine and sonicated for 10 min. Then the Co_3_O_4_/Oleylamine mixture was rapidly injected into 25 mL of oleylamine in a three-neck flask at 603 K under N_2_ atmosphere with continuous stirring. The reaction solution was maintained at 603 K for 20 min before being cooled down to room temperature. The product was precipitated with a mixture of hexane and ethanol and then collected via centrifugation. The reduced Co-based structures were obtained by extending the reaction time to 50, 80, and 120 min, respectively without changing the rest of the procedure.

### Materials characterizations and analysis

The crystal structure of the products was determined by room-temperature X-ray powder diffraction (XRD, Model D8 Advance, Bruker) with filtered Cu Kα radiation (λ = 1.5418 Å). The morphology and microstructure of the samples were characterized by a field emission scanning electron microscope (FE-SEM, HITACHI S−4800) equipped with energy dispersive X-ray spectroscopy (EDS). The internal microstructure of the products was characterized by a focused ion beam field emission scanning electron microscope (FIB-FESEM, Electron-Ion-Dual Beam System FEI Helios NanoLab 600i, Oxford INCA). Thermogravimetric analysis (TGA) was performed on a NETZSCH STA 449C thermogravimetric analyzer. The samples were heated in N_2_ atmosphere from room temperature to 673 K at 10 K·min^−1^ or heated in air from room temperature to 873 K at 5 K·min^−1^. Surface area of the samples was determined from the amount of N_2_ adsorption at 77 K. A Coulter Ominisorb 100cx, USA, was employed to obtain adsorption/desorption isotherms.

### Electrochemical characterization

The electrochemical measurements were carried out using a standard CR2032-type coin cell at room temperature. The working electrodes were prepared by pasting a slurry of Cu-based samples, carbon black (Super-P), and poly-(vinylidenedifluoride) (PVDF) with a weight ratio of 7:1:2 onto a piece of pure Cu foil. A pure lithium foil was used as the counter electrode, and a Celgard 2500 membrane was used as the separator. The electrolyte consisted of a solution of 1 M LiPF6 dissolved in a 1:1:1 mixture of ethylene carbonate (EC), ethylene methyl carbonate (EMC), and dimethyl carbonate (DMC). The cells were assembled in a glove box filled with high purity Ar gas. The galvanostatic discharge–charge experiments were performed over a voltage range of 0.02–3.0 V (vs. Li^+^/Li) at 0.2 C using a NEWARE BTS (Shenzhen, China) battery tester. All the capacities and C-rate currents were calculated based on active materials-Cu_2_O. Before tests of cycling performance, the test cells were activated for several cycles at 0.02 C.

## Additional Information

**How to cite this article**: Gao, L. *et al*. Synthesis of Hierarchical Nanoporous Microstructures via the Kirkendall Effect in Chemical Reduction Process. *Sci. Rep*. **5**, 16061; doi: 10.1038/srep16061 (2015).

## Supplementary Material

Supplementary Information
