# Supplementary material for: Synthesis of Hierarchical Nanoporous Microstructures via the Kirkendall Effect in Chemical Reduction Process
Source: Sci Rep. 2015 Nov 10;5:16061. doi: 10.1038/srep16061 (PMC4639846; doi:10.1038/srep16061)
Supplement: Supplementary Information [file srep16061-s1.doc]

**Synthesis of Hierarchical Nanoporous Microsctructures via the Kirkendall Effect in Chemical Reduction Process**

Ling Gao,^†^ Dafang He,^†^ Chao Pang,^†^ Liming Shen,^†^ Arunava Gupta,^‡^ Ningzhong Bao^†*^

^†^State Key Laboratory of Material-Oriented Chemical Engineering, Nanjing Tech University (Former Name: Nanjing University of Technology), Nanjing, Jiangsu 210009, P.R. China

^‡^Center for Materials for Information Technology (MINT), The University of Alabama, Tuscaloosa, AL 35487, USA

*Corresponding author:

Tel. & Fax: (+86)2583172244

E-mail: nzhbao@njtech.edu.cn (N. Bao)

**Content**

Figure S1--------------------------S2

Figure S2--------------------------S3

Figure S3--------------------------S3

Figure S4--------------------------S4

Figure S5--------------------------S5

Figure S6--------------------------S6

Figure S7--------------------------S7

Figure S8--------------------------S7

Figure S9--------------------------S8


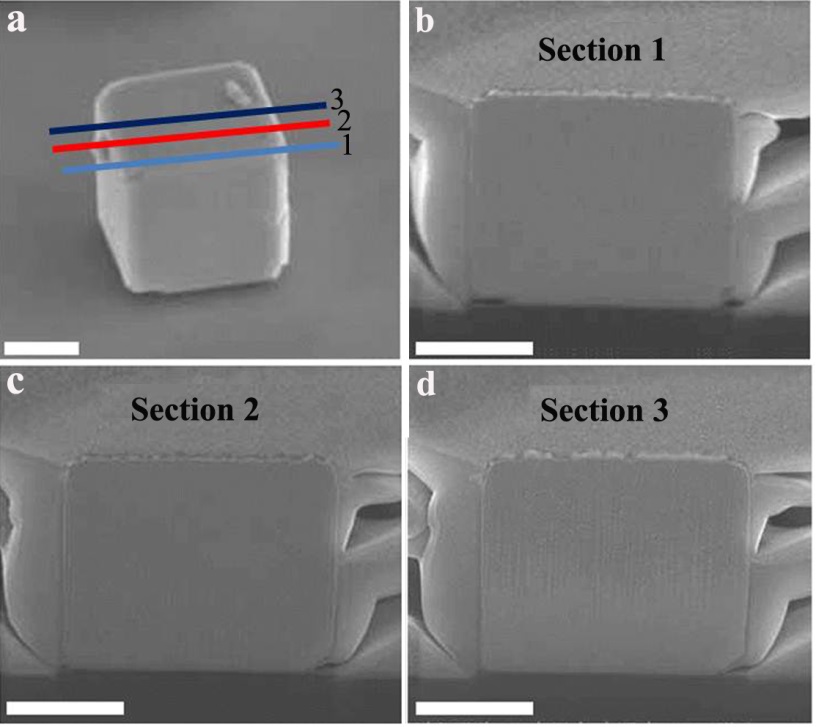


Figure S1. The inner structure of a non-porous Cu_2_O microcube. (a) FE-SEM image of a typical Cu_2_O microcube, showing the overall morphology. (b-d) FIB-FESEM images of the cross sections of the same sample cut along lines 1-3 marked in (a). All scale bars represent 500 nm.


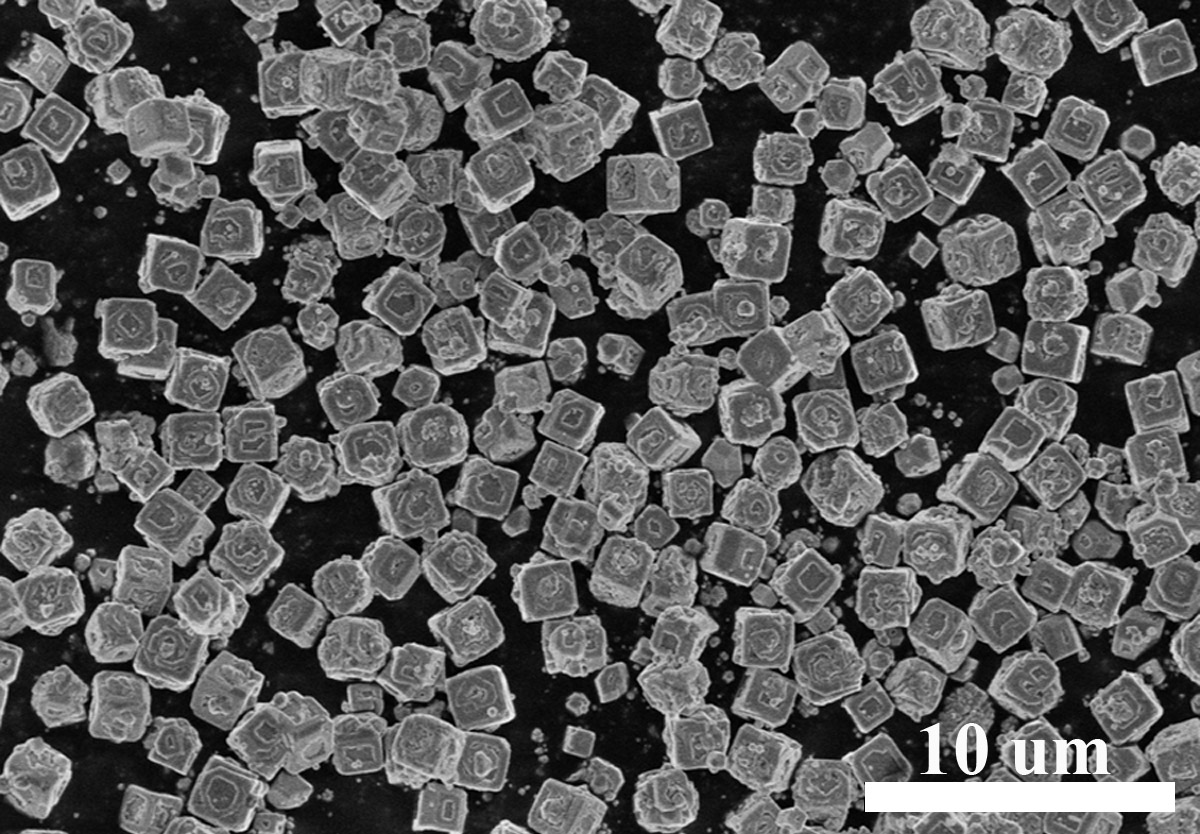


Figure S2. Large-area SEM image of nanoporous Cu/Cu_2_O/Cu dented cubic composites.


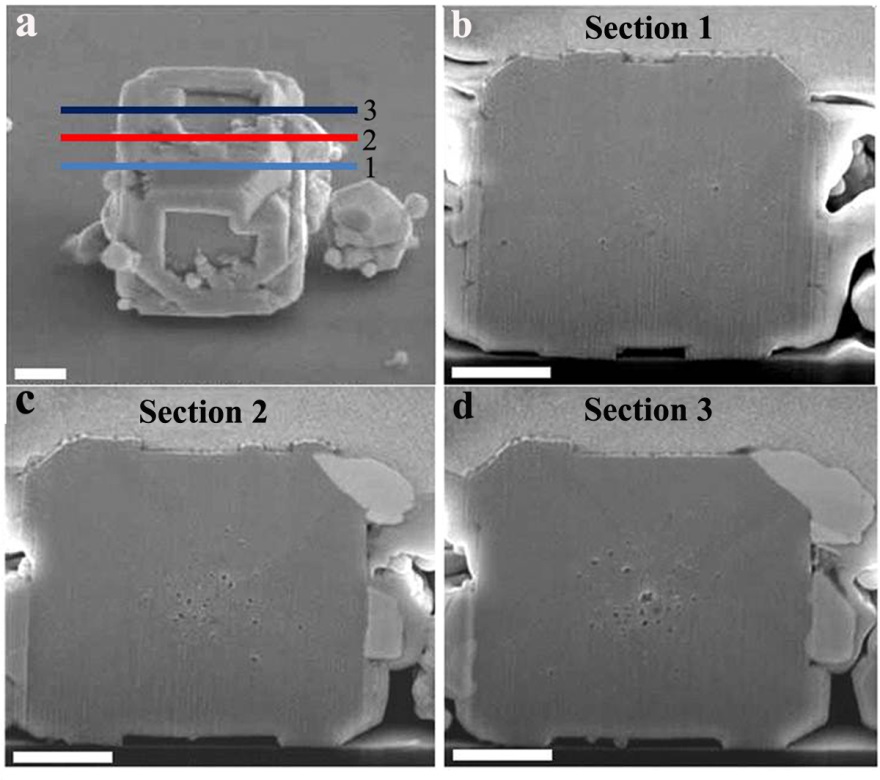


Figure S3. The inner structure of a nanoporous Cu/Cu_2_O/Cu dented cubic composite. (a) FE-SEM image of a typical Cu/Cu_2_O/Cu composite, showing the overall morphology. (b-d) FIB-FESEM images of the cross sections of the same sample cut along lines 1-3 marked in (a). All scale bars represent 500 nm.


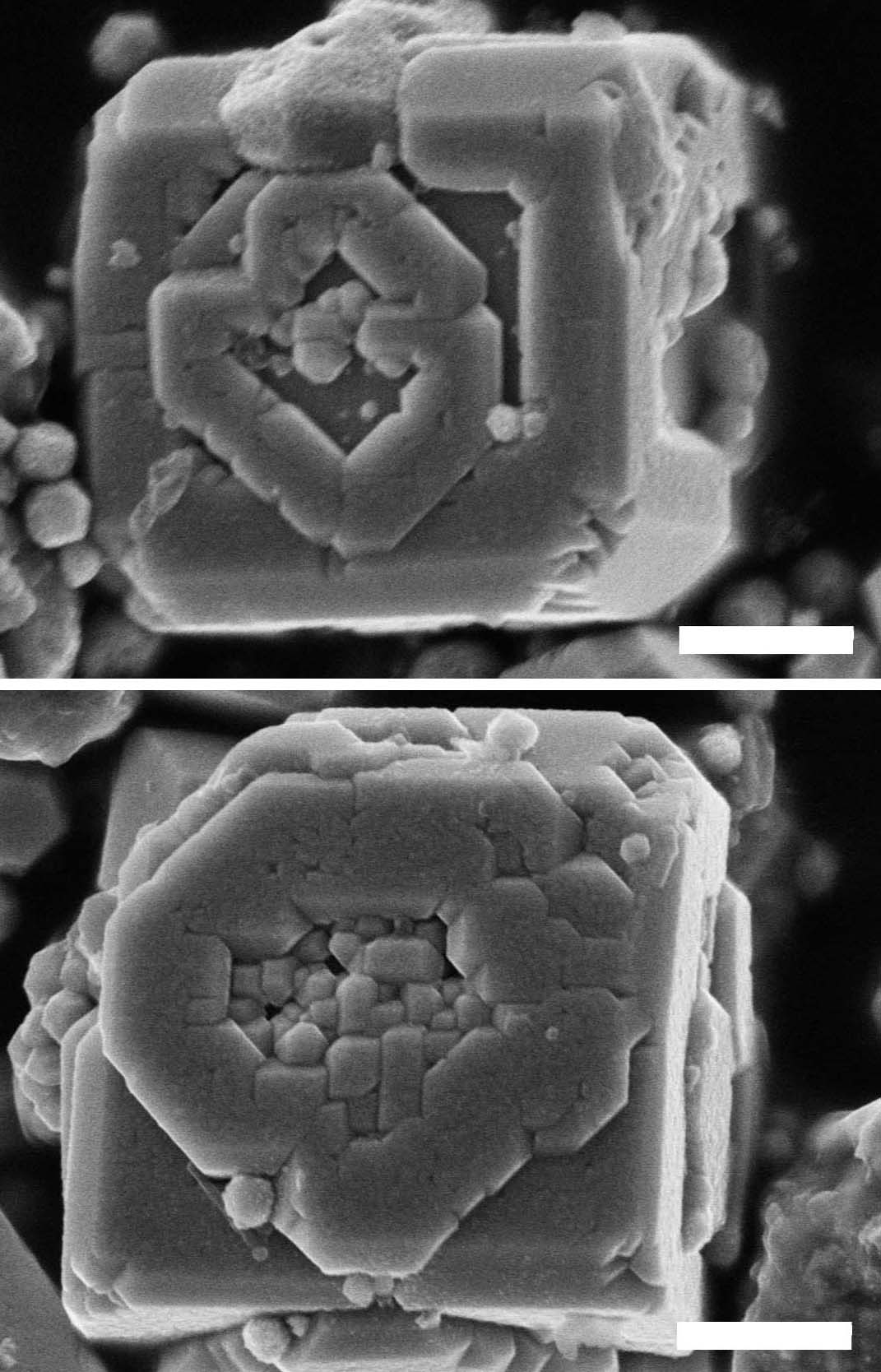


**Figure S4**. SEM images of the intermediate product of 50 min. The scale bars represent 500 nm.


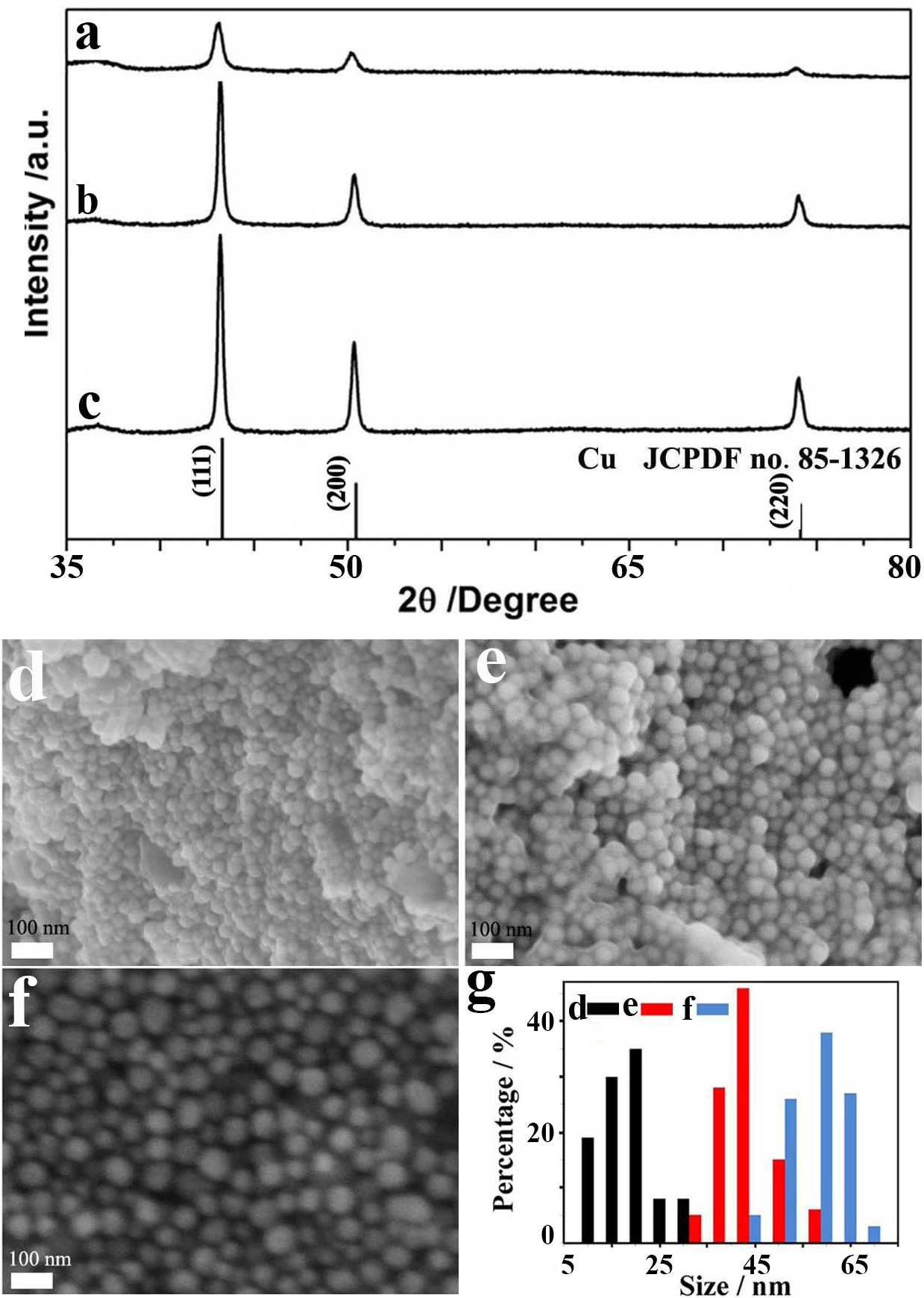


**Figure S5** (a-c) XRD pattern, (d-f) SEM images and (g) particle size distribution of the samples obtained by thermal decomposition of the Cu(CH_3_COO)_2_·H_2_O in oleic acid. Thermolysis condition is (a) (d) 543 K for 10 sec, (b) (e) 533 K for 10 sec and (c) (f) 533 K for 10 minutes. The scale bars represent 100 nm.


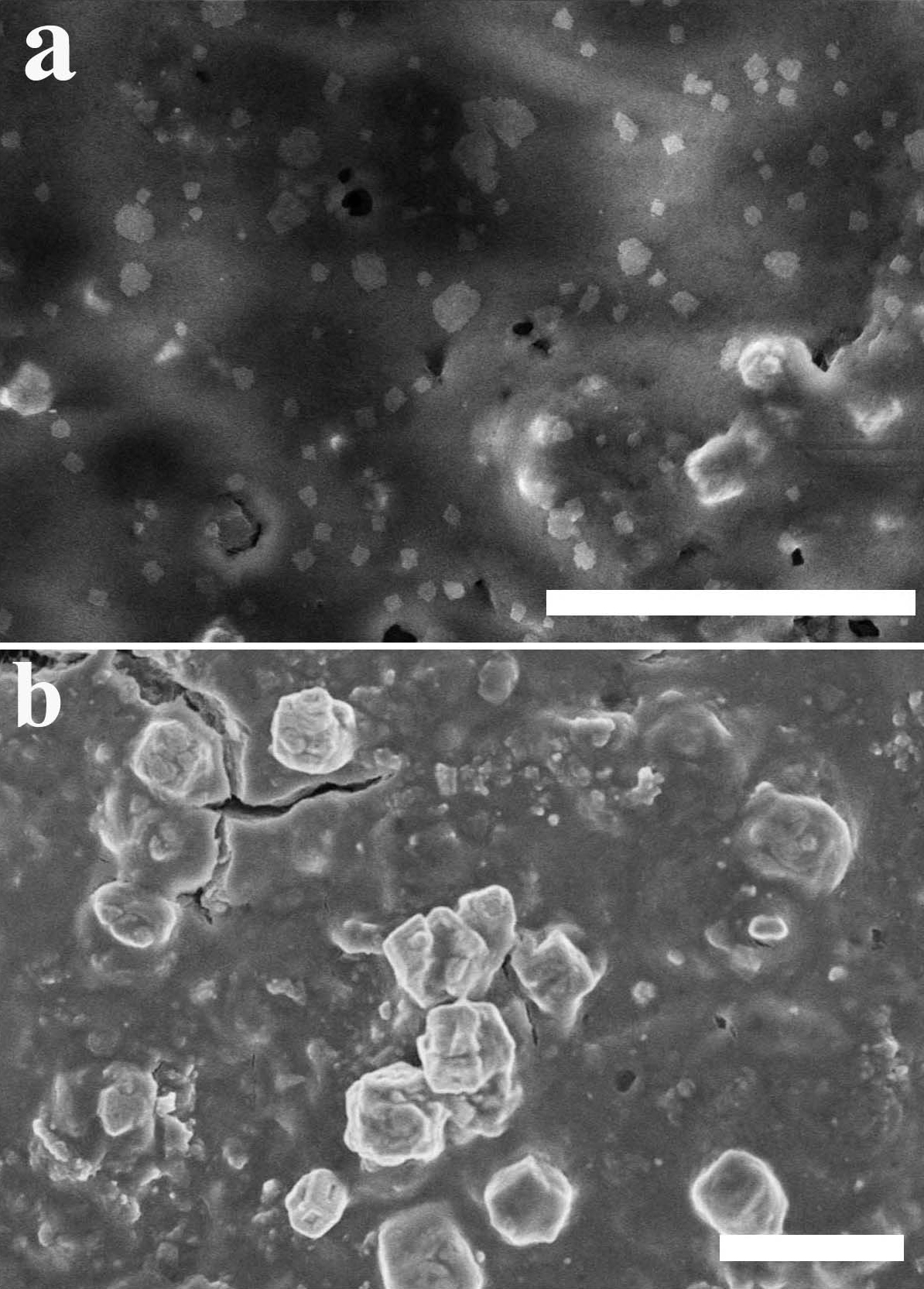


**Figure S6**. SEM images of the overall morphology of (a) solid non-porous Cu microcubes and (b) nanoporous Cu/Cu_2_O/Cu dented cubic composites after the after cycling test for Li-ion battery. The scale bars represent 5 um.


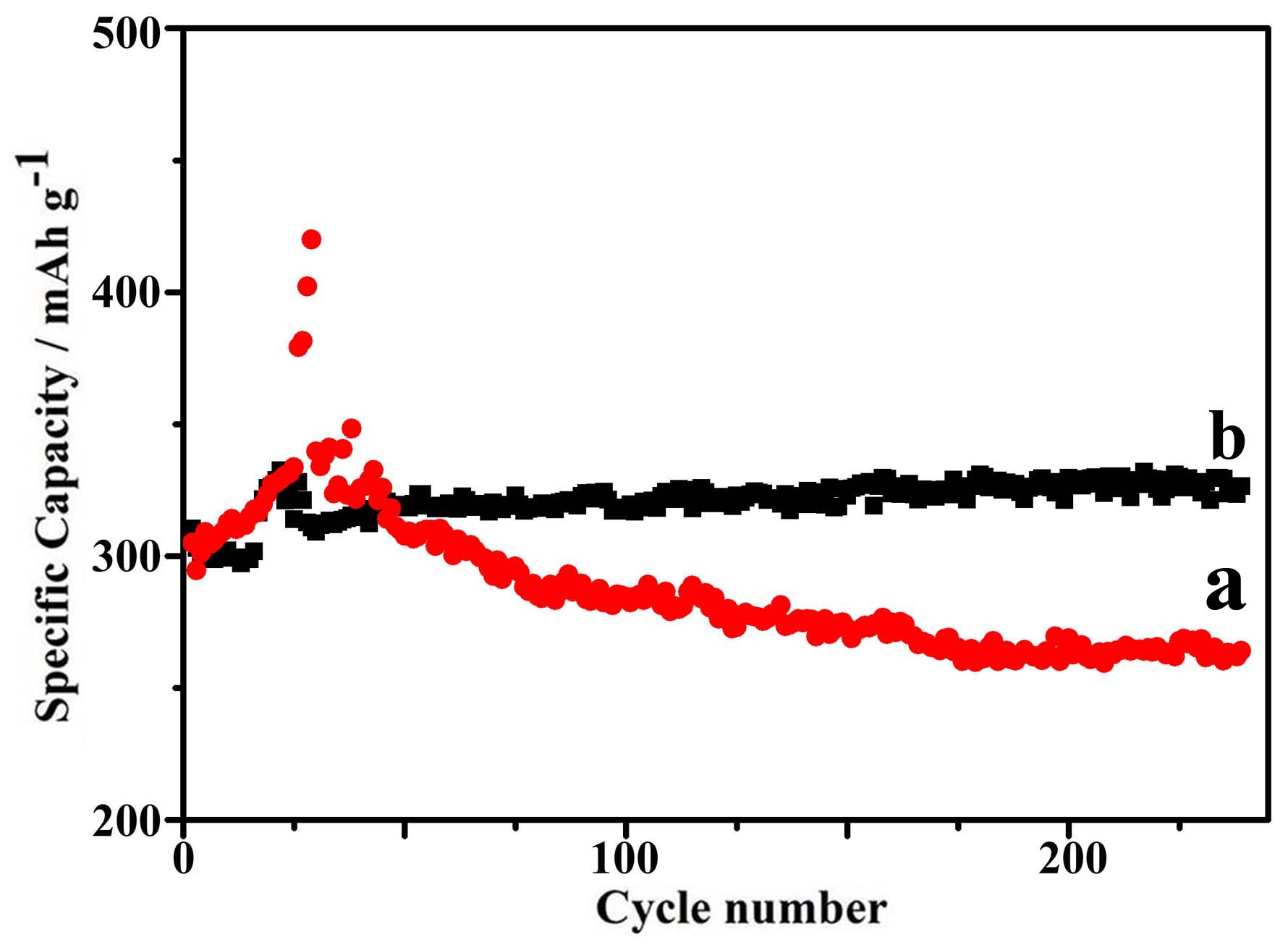


**Figure S7**. Cycling performances of Li-ion battery for the two different samples: (a) red for the solid non-porous Cu microcubes and (b) black for the nanoporous Cu/Cu_2_O/Cu dented cubic composites.


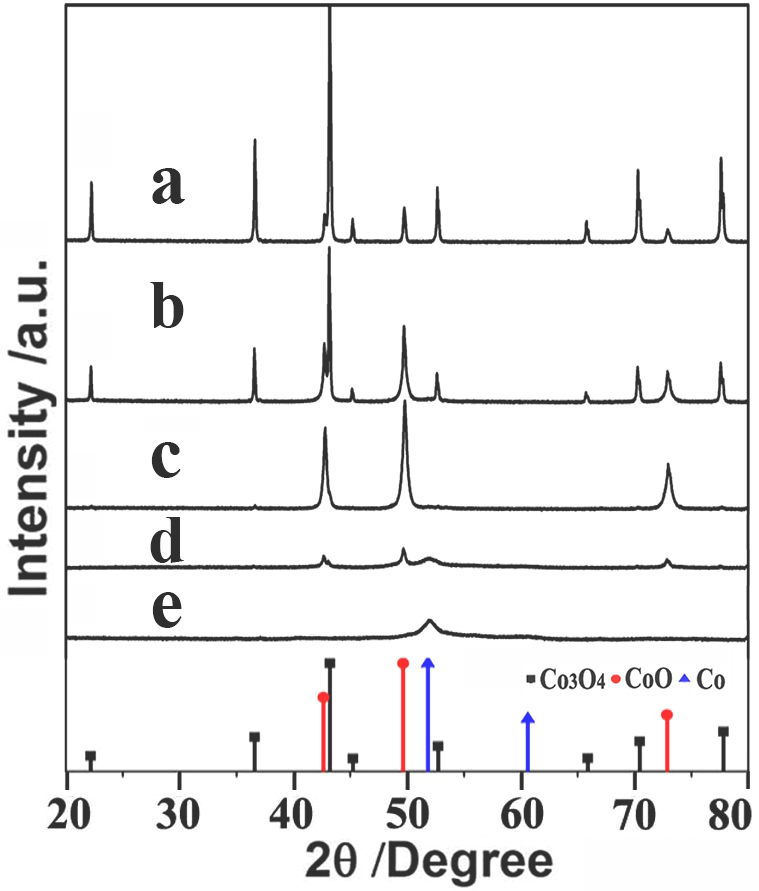


Figure S8. XRD patterns of (a) Co_3_O_4_, (b) Co_3_O_4_/CoO nanocomposites, (c) CoO, (d) CoO/Co, and (e) Co prepared by reducing Co_3_O_4_ in oleylamine at 603 K for 0, 20, 50, 80, and 120 min, respectively. The trace CoO impurity in the original Co_3_O_4_ particles is ignored because it does not influence the compositional and structural changes of products formed during the chemical reduction of Co_3_O_4_.


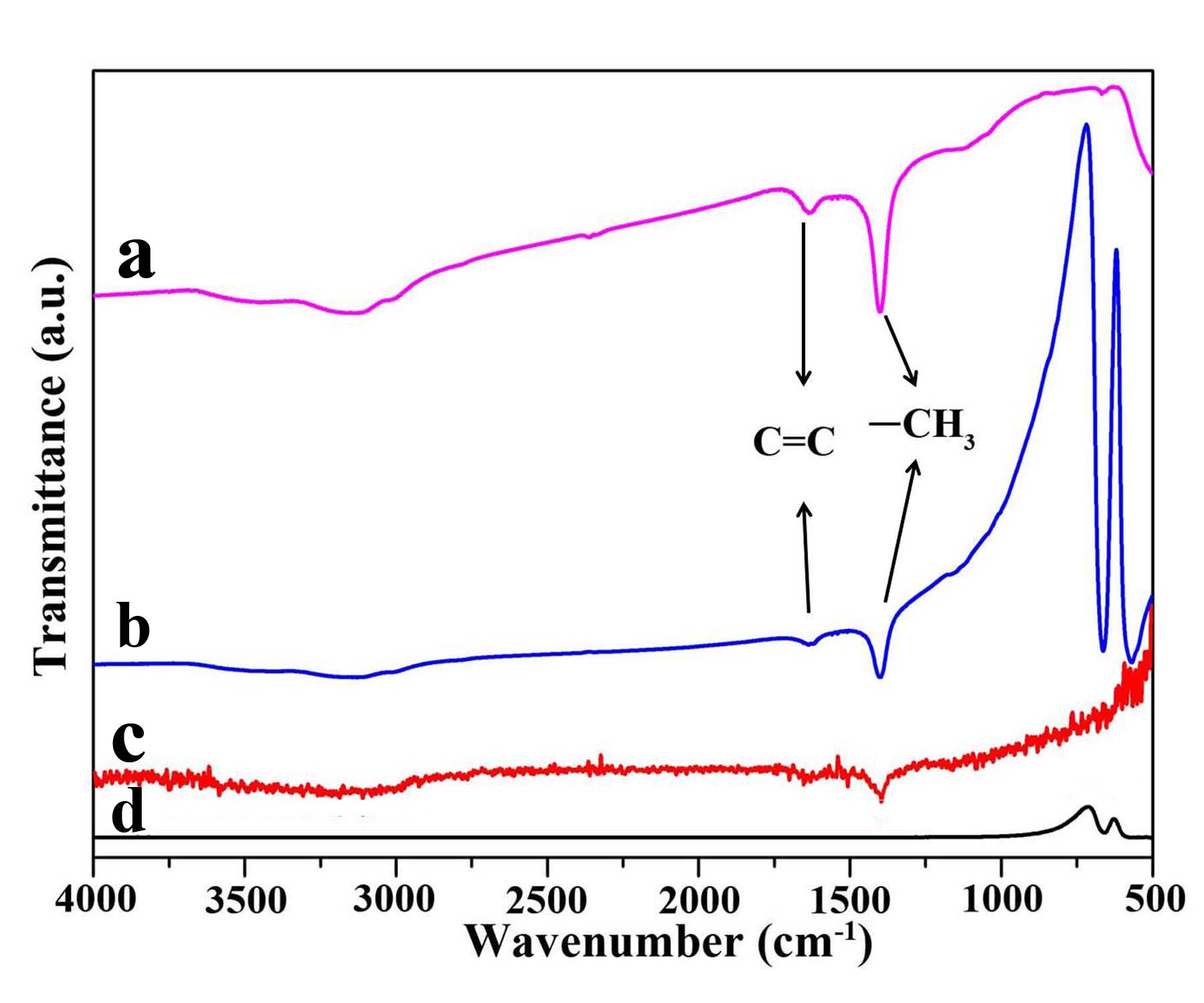


**Figure S9**. Fourier transform infrared spectra of the samples (a) Co particles calcined at 573 K, (b) (Co_3_O_4_/CoO) particles calcined at 573 K, (c) (Co_3_O_4_/CoO) particles without calcination and (d) the original Co_3_O_4_ particle.
